# Elementary Teacher's Perception of Oral Health Education in a City of the Brazilian Amazon: A Cross-Sectional Study

**DOI:** 10.1155/2024/8889140

**Published:** 2024-11-07

**Authors:** Samuel de Carvalho Chaves Junior, Deborah Ribeiro Frazão, Ângela Benedita da Costa e Silva, Nathalia Carolina Fernandes Fagundes, Roberta Souza D'Almeida-Couto, Márcio Antônio Raiol dos Santos, Rafael Rodrigues Lima

**Affiliations:** ^1^Laboratory of Functional and Structural Biology, Institute of Biological Sciences, Federal University of Pará, Belem, Pará, Brazil; ^2^School of Dentistry, Federal University of Pará, Belém 66075-110, Pará, Brazil; ^3^Center for Transdisciplinary Studies in Basic Education, Graduate Program in Basic School Curriculum and Management, School of Application, Federal University of Pará, Belem, Pará, Brazil

**Keywords:** cross-sectional study, education, oral health, schools

## Abstract

This study aimed to evaluate the level of oral health knowledge among teachers in a city of the Brazilian Amazon region (Belem, Pará) and explore the association between teachers' knowledge and their proposed actions in the event of dental trauma accidents within the school environment. The descriptive cross-sectional study involved 170 elementary teachers from public schools in the Metropolitan Region of Belem, Para State, Brazil. A self-administered questionnaire with multiple-choice questions was utilized to assess teachers' oral health knowledge across various domains. The questionnaire comprised seven targeted sections: the first section focused on demographic and professional aspects of the educators, while the remaining six sections addressed the oral health knowledge related to oral health education, dental caries, dental trauma (specifically dental avulsion), periodontal disease, visits to the dentist, and the educator's role in promoting oral health. Data analysis involved descriptive statistics and *χ*^2^ tests using Statistical Package for Social Science (SPSS; 20.0 version). Most of the sample were female (92.2%) and around 41–60 years (36.0%). Most teachers had over 5 years of teaching experience (87.0%) and held an undergraduate degree (63.0%). A significant association was found between teaching experience and the choice of storage method for avulsed teeth following dental trauma (*p*=0.005). However, the teachers examined in this study exhibited inadequate knowledge concerning the appropriate treatment for cavities and the recommended age for a child's first dental appointment. These findings underscore the crucial role teachers play in promoting oral health among students, particularly in regions characterized by social inequality and limited access to basic sanitation and healthcare services.

## 1. Introduction

According to the most recent national epidemiological study on oral health conducted in 2010 [1], caries has been recognized as the predominant oral disease in Brazil [1]. These results were concerning since they dropped significantly below of the World Health Organization's (WHO's) targets of 90% caries-free youngsters in 2010 and 100% by 2020 [2]. The northern area has the highest decaying, lost, and filled teeth index (SBBrazil 2010).

The results presented by the epidemiological study conducted in 2010 might be explained by the difficulties of obtaining dental health care [1]. The inadequate primary care coverage confirmed in various cities across this wide region indicates to a key aspect of the problem [3]. However, the issues are not limited to coverage and quality of care; there are additional factors, such as socioeconomic factors, lack of knowledge of parents, and insufficient education about oral health in schools [4].

Teachers play a significant role in facilitating the educational process and supporting children during their school years, more specifically in low-income regions such as the Brazilian Amazon, whose models of dental assistance targeted to the pediatric population embrace a curative approach and act only in biological determinants (e.g., microorganisms and diet). The lack of preventive approaches might result in the underdevelopment of good health habits and impaired oral health later in life [5].

The development of oral health promotion within the school environment may represent a great strategy to empower this population and improve their overall oral health in a long-term basis. It has been shown that oral health activities may improve the rate of good hygiene from 8.9% to 32% in preschool children [6]. Also, oral health promotion activities can have a high impact in society and low operating cost [7].

The importance of teaching children about dental hygiene has been recognized since 1878 [8]. During their formative years, children spend a significant amount of time in school, making it an ideal platform for promoting oral health, given its wide reach to over 1 billion children globally [9]. Primary school teachers play a crucial role in instilling healthy student habits. Studies have shown that teachers in Saudi Arabia possess substantial knowledge about oral health and are keen on promoting oral health education, highlighting their significant influence on students' overall well-being [10]. Integrating dental health education into the school curriculum and providing adequate training to teachers can further enhance their ability to foster good oral hygiene practices among students [11]. By recognizing oral health as an integral component of general health, teachers' awareness and dedication can positively impact students' habits, contributing to a healthier community overall [10].

In addition to promoting oral health, a thorough awareness of how to handle certain common problems in a child's life, such as dental trauma, is essential. Dental trauma has a substantial impact on a person's quality of life. A systematic review revealed that orofacial trauma had an immediate and increasing impact on oral health-related quality of life (OHRQoL) with the severity of the damage. Thus, health education campaigns must include information regarding the cause, prevention, and the importance of fast reactions from the public in accessing dental treatments [12].

This study aimed to verify the degree of buccal health knowledge of basic education, according to the level of schooling and time of experience in the teaching career. Moreover, this research can serve as an indicator for improving the teaching education in oral health and the insertion of the theme during the period of professional qualification.

## 2. Material and Methods

### 2.1. Study Design

This cross-sectional study was approved by the Ethics and Human Research Committee of the Institute of Health Sciences of the Federal University of Para, under the approval numbers 504.962 and 230.897. Additionally, the study was conducted in accordance with the Strengthening the Reporting of Observational Studies in Epidemiology (STROBE) statement [13].

### 2.2. Population Characteristic

This study was conducted with teachers from initial elementary school I, of any sex or gender, assigned as educators in the public schools in the Metropolitan Region of the city of Belem, Para State, Brazil. In Brazil, primary education is divided into elementary school I and elementary education II. Elementary education I comprise from Grades 1 to 5, also called initial series. Elementary education II goes from Grades 6 to 9 [14].

The study employed specific eligibility and exclusion criteria to ensure the relevance and focus of the research. Public school teachers in the initial grades of elementary school, actively teaching and serving in a regency position, were included. Conversely, those not currently teaching or in an administrative role were excluded. The data collection process involved visits to public schools offering elementary education in the Metropolitan Region of Belem, Brazil. Prior authorization from school coordinators was obtained to administer the questionnaire to teachers.

The sample size was calculated based on a universal population of 275 teachers. This represents the number of teachers in class leadership in the initial grades of elementary school I of the city. A 95% confidence level and 4% error margin were adopted, considering the power of 80%. In Belem and its metropolitan area, there is a total of 110 public schools as reported by the Instituto Brasileiro de Geografia e Estatística (IBGE) in 2021 [15]. To ensure a diverse representation, we randomly selected 22 schools based on their funding source, since public schools operate under different budgets depending on whether they are funded by the federal, state, or municipal/local government. Subsequently, all teachers from each of the selected schools were invited to participate in our study.

### 2.3. Questionnaire to Evaluate the Teachers' Knowledge

The questionnaire elected to meet the research objectives was a self-applicable questionnaire, with 18 multiple-choice questions. The instrument was designed with meticulous attention to detail, comprising seven distinct sections to comprehensively assess oral health knowledge among educators. The first section had questions (1) about the educator, focused on demographic (age and sex) and professional (schooling and work experience) aspects. Subsequent sections explored various facets of oral health, including (2) oral health education, (3) dental caries, (4) dental trauma of dental avulsion type, (5) periodontal disease, (6) the visit to the dentist, and (7) the role of educators in promoting oral health. To mitigate potential sources of bias, rigorous measures were implemented. First, the questionnaire was administered in the presence of researchers during school visits to ensure accuracy and consistency in responses. Additionally, efforts were made to control for potential confounders by considering factors such as socioeconomic status, educational background, and geographical location, which could influence participants' responses and perceptions of oral health. These strategies aimed to enhance the validity and reliability of the study findings, providing a robust foundation for analysis and interpretation (Data S1).

### 2.4. Statistical Analysis

Descriptive statistics were assessed, according to sociodemographic aspects, time of performance in the teachers, and schooling level of teachers. For the comparison of oral health knowledge, according to the degree of schooling (presence or absence of undergraduate degree, master's, or doctoral degree) and the work experience (up to 5 years or above 5 years of teaching), the *χ*^2^ test, with a significance level of 5%. The data collected were analyzed through the software Statistical Package for Social Science (SPSS; 20.0 SPSS Inc., Chicago, IL, USA).

## 3. Results

Out of the total of 232 educators who received an invitation to participate in the study, 62 of them declined to take part in it, while the remaining 170 teachers agreed to participate and were included in the study sample. Among the individuals who refused participation, a large part claimed a lack of time or disinterest in answering the questionnaire.

In Table 1, it is observed that 155 of the sample was female (91.2%). Most of the teachers were between 41 and 60 years old (36.0%), had more than 5 years (87.0%) of work experience, and had a graduate degree (63.0%).

Table 2 shows the distribution of the oral health knowledge variables according to both service time and schooling degree. A statistically significant difference was observed in the age at the first dental visit between teachers with and without a graduate degree (*p*=0.001). Additionally, there was a significant difference in the teacher's years of service and their level of education based on the storage method of the avulsed tooth following dental trauma (*p*=0.005).

## 4. Discussion

To the best of our knowledge, this study is the first to assess the pediatric oral health practices and knowledge of teachers in elementary schools from the Brazilian Amazon. The findings revealed that, for most variables analyzed, there were no differences in practices and knowledge among teachers based on their level of education or years of service. However, variations were observed in the handling of avulsed teeth and the recommended age for a child's first dental appointment.

Located in the Brazilian Eastern Amazon, Belem is the second most populous city in northern Brazil, characterized by social inequality and limited access to basic sanitation and healthcare services (IBGE, 2021). Most schools in the area do not have dentists or oral health professionals integrated into the school community for oral health promotion activities. As a result, teachers are often responsible for promoting oral health among students, as mandated by law [16].

According to the data from the National Institute for Educational Studies and Research (INEP), the number of teachers working in basic education totals about 1,939,000 in Brazil. Among these, in preschool education, there are 593,000, of which 79.1% have a university degree. In primary education, there are 793,000, of whom 91.8% have a university degree. Regarding the undergraduate level, 43.4% of the teachers have a postgraduate degree [17]. In the current study, the percentage of graduate teachers was very similar to the national data, with a slightly higher frequency.

Graduate teachers in this study indicated that the first dental appointment should occur as soon as possible, which differed from those without a higher education level. The results of this study suggest that possessing a graduate degree may be a distinguishing characteristic in the acquisition of knowledge regarding oral health. Furthermore, as individuals progress to advanced academic levels such as master's or doctoral degrees, they accumulate knowledge that can enhance their performance in the field of education [18].

Education encompasses transformative processes throughout an individual's life, guided by principles of autonomy and human interconnectedness. It is a shared responsibility of families and society. Educational institutions play a crucial role in implementing oral health programs as they bring together children in age-appropriate groups, facilitating the adoption of educational and preventive measures [19, 20].

The period of childhood is widely recognized as a critical phase for establishing long-term oral health outcomes [20]. In this study, a significant number of teachers indicated the importance of early dental appointments for children. It is during childhood that notions of oral health care habits are initiated and solidified, paving the way for future educational interventions to reinforce established routines [21, 22]. However, to effectively strengthen these habits, it is crucial to foster their development in an appropriate school environment.

School-age children are highly receptive and capable of learning, making this stage ideal for instilling appropriate habits, particularly those related to oral health. Moreover, teachers within the basic teaching network have a significant influence on students' behavior. Through daily interactions, methodological expertise, and establishing positive relationships, teachers can actively engage and motivate students in the development of good oral health habits. They act as valuable partners in preventive and educational programs [23].

It is essential that teachers possess adequate knowledge regarding the subject matter while participating in oral health activities with the school community. Recent studies have indicated that educators possess a certain level of familiarity with oral hygiene materials. However, their understanding of teeth caries and periodontal disease appears to be inadequate. Nevertheless, it is imperative that educators undergo training to effectively identify potential deviations from the norm in the child's stomatognathic system, as they are the primary professionals who will interact extensively with the child [24, 25]. The teachers examined in this study demonstrated inadequate knowledge regarding the appropriate treatment for avulsed teeth. Dental avulsion is a common injury among children in school environments and can lead to irreversible consequences, particularly in children whose oral structures are still developing. Hence, it is crucial to comprehend emergency protocols and adhere to appropriate methods for the storage of avulsed teeth [26–28].

The limited knowledge regarding the management of dental trauma in school environments has a profound impact on the training of future professionals who will be responsible for overseeing the well-being of children in schools [29]. This issue is even more concerning in the Amazon region, where access to urgent dental services can be challenging in certain areas. Empowering these professionals with the necessary knowledge and skills becomes crucial in such contexts.

Considering the limited knowledge on oral management from the teachers and absence of integrated dental professionals in schools, the incorporation of innovative approaches like virtual reality (VR) holds the potential to address educational disparities. VR has been acknowledged for its efficacy in dental education, providing a simulated environment conducive to fine motor skill training and the enhancement of hand–eye coordination [30]. The integration of VR into oral health programs tailored for teachers could offer a dynamic and engaging platform, fostering a deeper comprehension of oral health practices, and responding to emergencies such as the management of avulsed teeth. However, it is essential to acknowledge that widespread adoption of this technology in schools within the Metropolitan Region of Belem would require substantial funding. Consequently, research projects initiated by public universities become imperative, as direct school funding may prove insufficient to cover the costs associated with implementing a VR approach [31].

Some limitations should be acknowledged in this study. First, the final number of participants fell short of the initially calculated sample size, although the study still had a statistical power of 80%. Additionally, since the questionnaire was self-administered, there is a possibility of overreporting or underreporting of answers. Moreover, the sample consisted solely of teachers from public schools in a single city within the Brazilian Amazon, indicating the need for a more diverse and representative sample from various regions and types of schools in Brazil.

This study revealed a concerning lack of knowledge among basic public school teachers in the Amazon region of Brazil regarding caries, periodontal disease, and dental avulsion. Many teachers also reported not regularly engaging in oral health activities with children. However, in an area where teacher participation is crucial for oral health promotion, government initiatives are needed to provide training on oral health topics and incorporate them into teachers' educational programs. These measures are vital for equipping teachers with the necessary information and effective techniques, ultimately contributing to the improvement of children's oral health outcomes.

## 5. Conclusion

The study evaluates oral health practices and knowledge of teachers in Brazil's Amazon region, revealing no significant differences based on educational background or teaching experience. Enhancing teachers' knowledge and training on dental trauma management and oral health promotion would have significant benefits, especially in regions with limited access to emergency dental services.

## Figures and Tables

**Table 1:** Characterization regarding the sociodemographic and professional of the teachers of the initial series of elementary school of public schools in Belém, Brazil, 2014.

Sociodemographic and professional aspects	*n* (%)
Sex	
Female	155 (91.2)
Male	15 (8.8)
Age	
20–35	29 (17.0)
36–45	49 (28.8)
46–60	61 (36.0)
Above 60	31 (18.2)
Service time	
Up to 5 years	22 (13.0)
Above 5 years	148 (87.0)
Postsecondary degree	
Yes	107 (63.0)
No	63 (47.0)

**Table 2:** Difference between service time and schooling degree of elementary teachers at public schools in the city of Belém, Brazil, in relation to knowledge and oral health practices.

Independent variables	Service time					Schooling degree				
	Up to 5 years *n* = 21		Above 5 years *n* = 149		*p* value*⁣*^*∗*^	Without graduate *n* = 63		Graduate *n* = 107		*p* value*⁣*^*∗*^
	*n*	%	*n*	%		*n*	%	*n*	%	
Sources of information on oral health										
Up to 2 sources	15	68.1	85	55.4	0.309	33	50.8	67	60.7	0.250
3–5 sources	6	31.9	64	44.6		30	49.2	40	39.3	
Activities in oral health										
Yes	8	63.6	49	33.1	0.828	27	57.1	30	72	0.0705
No	13	36.4	100	66.9		36	42.9	77	28	
Knowledge about caries										
One correct alternative	5	22.7	44	22.9	0.584	15	15.9	34	24.3	0.223
Two correct alternatives	6	22.7	55	29		28	31.7	33	23.4	
Three correct alternatives	6	36.4	24	16.8		8	17.5	22	18.7	
Four correct alternatives	4	18.2	26	31.3		12	34.9	18	33.6	
Preventive materials for caries lesions										
Toothbrush/dental floss/toothpaste	3	13.6	22	10.8	0.886	10	11.1	15	11.2	0.093
Toothbrush and toothpaste/toothbrush and dental floss/dental floss and mouthwash	4	22.7	25	15.5		5	12.7	24	8.4	
Toothbrush, toothpaste, and dental floss/toothbrush, mouthwash, and dental floss	3	9.1	32	21		15	20.6	20	18.7	
Toothbrush, toothpaste, dental floss, and mouthwash	11	54.6	70	52.7		33	55.5	48	60.7	
Knowledge about the characteristics of periodontal disease										
One correct alternative	6	27.2	50	32	0.888	18	23.8	38	33.6	0.060
Two correct alternatives	6	27.2	29	19.6		14	23.8	21	14	
Three correct alternatives	1	9.1	22	14.1		7	11.1	16	15.9	
Four correct alternatives	1	4.5	6	4		4	6.3	3	16.8	
Answered all	0	0	2	2		1	1.6	1	0.9	
Do not know	7	32	40	28.3		19	33.3	28	18.7	
Encourages dental appointment										
Yes	12	59	115	77	0.122	43	68.3	84	78.5	0.141
No	9	41	34	23		20	31.7	23	21.5	
Indicated age for first dental appointment										
Before deciduous teeth eruption	11	50	63	44	0.728	30	50.8	44	41.1	0.001*⁣*^*∗*^
First deciduous tooth eruption	3	18.2	40	28.4		8	15.9	35	34.6	
Deciduous dentition	4	18.2	15	14.1		4	7.9	15	9.3	
First permanent tooth eruption	2	9.1	15	8.8		11	15.9	6	9.3	
Permanent dentition	1	4.5	16	4.7		10	9.5	7	5.6	
Tooth storage after dental avulsion										
Out	14	68.2	80	54	0.028*⁣*^*∗*^	33	52.4	61	57	0.005*⁣*^*∗*^
Watery	1	4.5	17	12.8		1	3.2	17	19.6	
Saliva	2	9.1	0	1.4		1	0	1	2.8	
Do not know	4	18.2	52	31.8		28	44.4	28	20.6	

*⁣* ^*∗*^ *χ* ^2^ test *p*  ≤ 0.05.

## Data Availability

The authors confirm that the data supporting the findings of this study are available within the article and its Supporting Information.

## Supporting Information

Supporting Information Data S1: STROBE Statement—checklist of items that should be included in reports of observational studies.
